# 

**Published:** 1874-08

**Authors:** 


### Table of Contents.

### ORIGINAL COMMUNICATIONS.

OVARIOTOMY—Its Relations to Septicæmia, Pyæmia, etc. *By Prof. J. T. Gilmore, M.D., Mobile, Ala.* ........ 257
SACCHARATED CALOMEL. *By John M. Johnson, M.D., Atlanta, Ga.* ....... 277
UTERINE TUMOR—CASE. *By A. Couvert, M.D* ........ 286
SACCHARATED CALOMEL. *By W. H. Saxon, M.D., Dirt Town, Ga.* ....... 288

### MEDICAL SOCIETIES.

ATLANTA ACADEMY OF MEDICINE—Ligation of Arteries for Traumatic Inflammation—Dislocation Hip, of ten weeks' standing—Trephining Skull for Epilepsy—Paralysis of *portio dura*—Pirogoff's Operation—New Intra-Uterine Stem - Ipecac Treatment in Dysentery—Normal Ovariotomy, another case--Ovulation and Menstruation.. 288

### OUR EXCHANGES.

Rusticus in Town (poetical) .... 295
Chronic Cystitis—drainage .... 297
Cancer Cures—formulæ .... 299
Quinine Pill Mass—formulæ .... 300
"The Doctor" ....
Jarborandi, a New Medicine .... 301
A College for Specialties .... 302
Strychnia in Whisky .... 303
Electrolysis Extraordinary ....
Croton Chloral Hydrate .... 304
Tractile Method in Strangulated Hernia .... 305
Laparotomy, Remedy for Intussusception .... 306
Penetrating Wounds of Great Cavities .... 307
Lumbar Colotomy for Obstruction of Rectum ....
Rheumatism, Cerebral Embolism, Paralysis, Recovery .... 308
Chronic Vaginitis—Treatment .... 309
Obstetric Aid .... 313

### BIBLIOGRAPHICAL.

Ligation of Arteries—Farabeuf ....
College Announcements ....
Pamphlets Received .... 311
New Journals ....
Materia Medica for Students—Biddle .... 312

### EDITORIAL.

Is there any thing New under the Sun? .... 314
Can any Good Thing come out of Nazareth? ....
Indian Spring .... 318
Secular Press and Medical Advertisements ....
Novel Remedy for Whooping-Cough .... 319
Acknowledgments .... 320

